# Tumefactive Multiple Sclerosis Mimicking an Intracranial Mass in a 34-Year-Old Man: A Case Report

**DOI:** 10.7759/cureus.105046

**Published:** 2026-03-11

**Authors:** Zaid Al Hassani, Razan Darwish, Bashar Al Samirraie, Mustafa Aal Yaseen, Areen Said

**Affiliations:** 1 Medicine, University of Sharjah, Sharjah, ARE; 2 Neurology, American Hospital, Dubai, ARE

**Keywords:** atypical multiple sclerosis, case report, inflammatory demyelinating disease, oligoclonal bands, ring-enhancing intracranial lesion, tumefactive multiple sclerosis

## Abstract

Tumefactive multiple sclerosis (TMS) is a rare demyelinating variant of multiple sclerosis that can closely mimic intracranial malignancy or abscess on imaging. This case highlights diagnostic complexity when focal neurological deficits occur alongside a concurrent dermatomal rash.

A 34-year-old man with no prior neurological history presented with three days of progressive left-sided weakness and numbness, preceded by ipsilateral neck pain and a pruritic vesicular rash, initially treated as herpes zoster. Brain imaging revealed a 3.5 cm right frontal ring-enhancing lesion with surrounding vasogenic edema and minimal mass effect. Cervical spine MRI demonstrated a concurrent enhancing lesion at C2 to C3. Cerebrospinal fluid showed mildly elevated protein and oligoclonal bands, supporting an inflammatory demyelinating process. He was treated with intravenous methylprednisolone (1 g daily for five days) followed by an oral prednisone taper, with complete clinical recovery at four-week follow-up. Brain biopsy was deferred after neurosurgical consultation.

Integrating clinical context, MRI patterns, and CSF findings can support early recognition of TMS and help avoid unnecessary antimicrobial therapy or invasive diagnostic procedures.

## Introduction

Multiple sclerosis (MS) is a chronic central nervous system disorder characterized by demyelination, inflammation, and axonal injury, often occurring in relapsing episodes [1]. MS is more common in females than males, and onset most frequently occurs between 20 and 40 years of age [2].

Although typical MS presents with small multifocal demyelinating plaques, rare variants such as tumefactive multiple sclerosis (TMS) present distinct diagnostic challenges. TMS is a rare MS variant defined by large demyelinating lesions, typically greater than 2 cm, that may demonstrate surrounding edema, mass-like appearance, and ring enhancement, thereby mimicking neoplasm, abscess, or other inflammatory conditions [3,4]. Reported incidence is low, estimated at 0.3 to 0.7 cases per 100,000 individuals annually, with a modest female predominance (male-to-female ratio approximately 1:1.3), making presentations in male patients relatively less common [5]. Clinical manifestations depend on lesion location and can include headache, cognitive impairment, confusion, aphasia, apraxia, seizures, and focal neurological deficits [4].

The pathophysiology of TMS is incompletely understood, but histopathology typically shows confluent demyelination with reactive astrogliosis and relative axonal preservation compared with conventional MS [4,6]. Neuroimaging is central to diagnosis, and features that may support TMS over common mimics include incomplete ring enhancement and limited mass effect relative to lesion size, alongside supportive clinical and cerebrospinal fluid (CSF) findings [1,3]. Magnetic resonance (MR) spectroscopy may further aid differentiation by demonstrating elevated choline without lactate or lipid peaks that may be seen in necrotic tumors [7,8]. CSF assessment provides important supportive evidence, as oligoclonal bands are commonly present, although their absence does not exclude TMS [4,9]. When imaging and CSF findings are concordant, current practice generally favors avoiding brain biopsy and initiating immunosuppressive therapy, most commonly high-dose corticosteroids; escalation therapies may be required in steroid-refractory cases [1,5,10,11].

We report a 34-year-old man who presented with progressive unilateral weakness and sensory deficits preceded by a dermatomal vesicular rash initially suggestive of herpes zoster, and was found to have a ring-enhancing right frontal lesion with a concurrent cervical cord lesion and CSF oligoclonal bands. This case highlights how careful clinicoradiological correlation and CSF evaluation can support early recognition of TMS and help avoid unnecessary antimicrobial therapy or invasive diagnostic procedures.

## Case presentation

A 34-year-old man with no prior neurological history presented to the emergency department with a three-day history of progressive left-sided weakness and numbness. Approximately two days before the onset of neurological symptoms, the patient initially reported burning discomfort on the left side of the neck radiating to the shoulder and upper arm, accompanied by a pruritic vesicular rash in a C3 to C4 dermatomal distribution. He was initially diagnosed with herpes zoster and started on valacyclovir. The diagnosis was made clinically based on the characteristic dermatomal vesicular rash, and confirmatory PCR testing or viral swab was not performed. Despite antiviral therapy, his neurological symptoms progressed, prompting further evaluation. He denied visual symptoms, balance disturbance, or cognitive changes. There was no family history of multiple sclerosis or other autoimmune disease. He had no known chronic medical conditions, was not taking regular medications, and reported no drug allergies. He denied tobacco, alcohol, and recreational drug use. There was no relevant travel history, toxin exposure, or recent vaccination. At baseline, he was independent in activities of daily living.

Vital signs were within normal limits, and he was afebrile. Cranial nerve examination was normal, and there were no meningeal signs. A pruritic vesicular rash with erythematous papules was present on the left lower neck extending to the mid-back, consistent with herpes zoster. Neurological examination demonstrated left-sided hemiparesis (Medical Research Council grade 4/5 in both the upper and lower limbs), reduced light touch and pinprick sensation over the left hemibody, mild dysmetria on finger-to-nose testing, and an ataxic gait with leftward veering.

**Figure 1:**
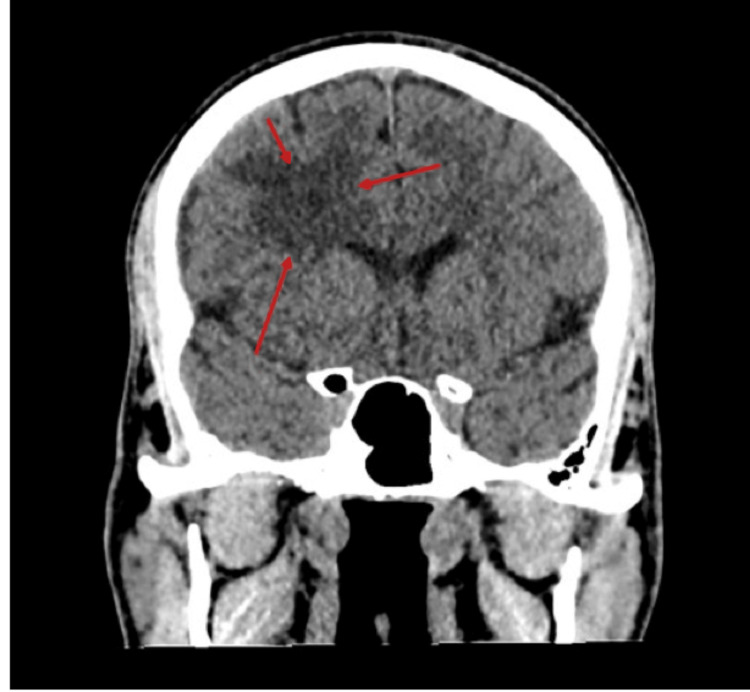
Non-contrast CT of the brain showing a right frontal hypodense lesion Non-contrast axial CT image shows a 3.5 × 3.0 × 2.6 cm hypodense lesion (arrow) in the right anterior frontal lobe white matter. No hemorrhage, significant mass effect, or midline shift is observed. This finding raised initial concern for a demyelinating or cystic process.

**Figure 2:**
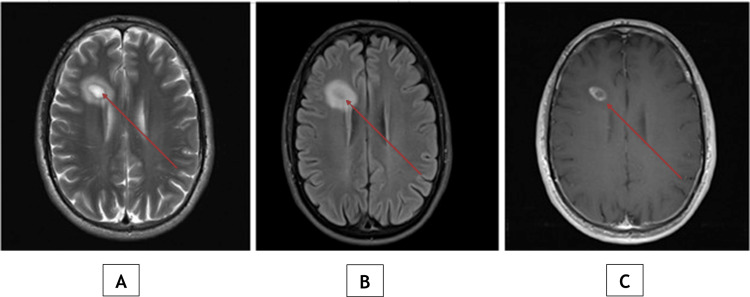
Contrast-enhanced brain MRI showing a ring-enhancing right frontal lesion with surrounding edema Axial T1-weighted post-contrast MRI (C) shows an irregular ring-enhancing lesion in the right frontal lobe (arrow) with heterogeneous peripheral enhancement and focally thicker margins. Surrounding vasogenic edema is seen as hyperintensity on T2-weighted (A) and fluid-attenuated inversion recovery (FLAIR) (B) sequences.

Non-contrast computed tomography (CT) of the brain (Figure 1) showed a 3.5 × 3.0 × 2.6 cm hypodense lesion in the right anterior frontal lobe white matter, without hemorrhage, significant mass effect, or midline shift. Brain magnetic resonance imaging (MRI) (Figure 2) demonstrated a ring-enhancing lesion in the right frontal lobe with surrounding vasogenic edema on T2-weighted and fluid-attenuated inversion recovery (FLAIR) sequences. Enhancement was heterogeneous, with focally thicker margins, and there was no significant mass effect. 

**Figure 3:**
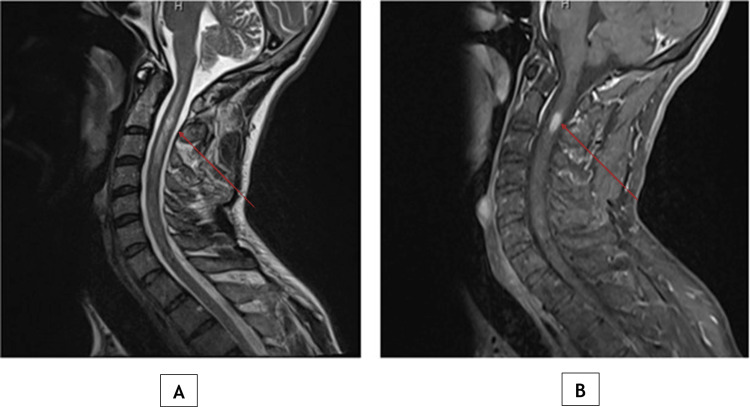
Cervical spine MRI showing an enhancing lesion at C2 to C3 consistent with demyelination Sagittal T2-weighted MRI (A) shows the arrow indicating a focal intramedullary hyperintense lesion in the cervical cord at C2-C3. Post-contrast sagittal T1-weighted MRI (B) shows the arrow indicating corresponding enhancement of the same lesion, consistent with active inflammatory demyelination. In conjunction with the intracranial lesion, these findings support the diagnosis of tumefactive multiple sclerosis.

Spinal MRI (Figure 3) demonstrated an enhancing lesion at the C2 to C3 level, consistent with inflammatory demyelination in the cervical spinal cord.

Advanced MRI techniques such as MR spectroscopy were considered in the diagnostic evaluation; however, spectroscopy was not performed, and the diagnosis was established based on conventional MRI features in conjunction with cerebrospinal fluid findings.

Initial laboratory investigations revealed a normal total white blood cell count with a normal differential leukocyte count and inflammatory markers, including C-reactive protein (CRP) and erythrocyte sedimentation rate (ESR), which did not suggest an active infectious process. HIV testing was also negative. These findings further reduced the likelihood of infectious or immunocompromised-related etiologies such as cerebral abscess or lymphoma.

Cerebrospinal fluid (CSF) analysis (Table 1) showed mildly elevated protein (0.49 g/L; reference range 0.18 to 0.45 g/L) and the presence of oligoclonal bands, supporting an inflammatory demyelinating process. CSF white cell count and glucose were within normal limits. Aquaporin-4 antibody testing was negative, making neuromyelitis optica spectrum disorder less likely. Cerebrospinal fluid varicella zoster virus (VZV) PCR testing was negative, and serum VZV serology was negative. 

**Table 1:** Cerebrospinal fluid (CSF) analysis

	Patient result	Reference range
Appearance/color	Colorless	Colorless
White cell count (cells/µL)	<5	<5
Glucose (mmol/L)	2.9	2.5-3.5
Protein (g/L)	0.49	0.18-0.45
Oligoclonal bands	Present	Absent

Differential diagnoses included tumefactive demyelination, pyogenic abscess, primary central nervous system (CNS) neoplasm, and metastasis based on the ring-enhancing frontal lesion with surrounding edema. However, the absence of fever, leukocytosis, and CSF pleocytosis made abscess less likely, while the relatively limited mass effect for lesion size and concordant cervical cord demyelinating lesion favored an inflammatory demyelinating process over neoplasm or metastasis. Targeted testing for tuberculosis, fungal infection, and toxoplasmosis was negative during this admission, and exclusion of these less likely infectious mimics was based on clinical stability, normal inflammatory markers, absence of CSF pleocytosis, HIV negativity, and imaging patterns that favored demyelination.

He received intravenous methylprednisolone (1 g daily for five days), followed by an oral prednisone taper. Neurosurgery was consulted because of the ring-enhancing lesion. After neurosurgical consultation, biopsy was deferred because the concordant MRI and CSF findings supported a demyelinating process, and the patient showed rapid clinical improvement after corticosteroid therapy. The lesion demonstrated ring enhancement with surrounding vasogenic edema and minimal mass effect on MRI, features that are characteristic of tumefactive demyelination. Additionally, spinal MRI revealed a concurrent demyelinating lesion at the C2-C3 level, and CSF analysis showed oligoclonal bands, further supporting an inflammatory demyelinating process rather than a neoplastic etiology.

At four-week follow-up, neurological examination showed full recovery, with strength improving to 5/5 and complete resolution of sensory symptoms, gait ataxia, and coordination deficits. No steroid-related adverse effects were reported. A repeat MRI was not performed at that time due to the full clinical resolution of symptoms. However, continued neurological follow-up was recommended to monitor for possible recurrence or progression to multiple sclerosis. Disease-modifying therapy was not initiated at this stage because this was considered a single demyelinating event with complete clinical recovery; it would be reconsidered if the patient develops recurrent episodes or fulfills diagnostic criteria for multiple sclerosis.

The patient reported significant anxiety about the possibility of a brain tumor and expressed relief after clinical improvement and clarification of the diagnosis. The primary diagnostic challenge was the combination of a ring-enhancing intracranial lesion and a concurrent dermatomal rash, which initially increased concern for an infectious etiology. Multimodal neuroimaging and CSF biomarkers were therefore prioritized to avoid unnecessary antimicrobial therapy or invasive diagnostic procedures.

## Discussion

Tumefactive multiple sclerosis (TMS) represents a diagnostic dilemma because it can resemble neoplastic or infectious intracranial lesions. While conventional multiple sclerosis lesions rarely exceed 1 to 2 cm, TMS lesions often measure more than 2 cm and may demonstrate mass-like features such as perilesional edema and ring enhancement [3,4]. This case underscores the importance of integrating clinical, radiological, and laboratory data to avoid misdiagnosis, particularly in less typical populations such as male patients [5].

TMS presents a diagnostic challenge because it can mimic brain tumors and abscesses on imaging and occurs infrequently. Many patients experience a monophasic clinical episode and do not progress to conventional multiple sclerosis [4]. Clinical manifestations vary by lesion location and may include headache, cognitive impairment, confusion, motor and sensory deficits, and cerebellar dysfunction [7]. Advanced MRI sequences can support differentiation: peripheral diffusion restriction in TMS, related to inflammatory cellular infiltration, contrasts with the central restriction typical of pyogenic abscesses, and MR spectroscopy may show elevated choline without the lactate or lipid peaks more suggestive of neoplasms [8].

In our case, the patient had left-sided motor and sensory deficits, and the diagnostic evaluation was complicated by preceding ipsilateral pain and a pruritic dermatomal rash suggestive of herpes zoster. This rash initially suggested herpes zoster infection and raised concern for a possible infectious or inflammatory trigger. However, the temporal association was ultimately interpreted as coincidental, although viral infections have occasionally been proposed as potential triggers of demyelinating events. TMS lesions most commonly occur in supratentorial white matter, including periventricular and subcortical regions, and can also involve the cervical spinal cord. Despite their size, they may demonstrate relatively limited mass effect with surrounding vasogenic edema. Imaging features that favor TMS over neoplasm or infection include incomplete ring enhancement, minimal mass effect relative to lesion size, and lack of frank necrosis, which were consistent with our patient’s imaging findings [3,7]. Compared with primary CNS lymphoma, TMS lesions are often hypointense on T1-weighted images and hyperintense on T2-weighted images [8]. In contrast, cerebral abscesses typically demonstrate central diffusion restriction due to purulent material, while high-grade tumors often show irregular thick ring enhancement with substantial surrounding edema and progressive mass effect. Clinical context also remains important in narrowing the differential diagnosis, including progressive deficits in glioblastoma, a known primary malignancy in metastasis, fever and leukocytosis in abscess, and immunocompromised or older age groups in lymphoma [3,7,8].

Because clinical and imaging features can overlap with infection and malignancy, additional diagnostic evaluation is often required, including CSF analysis. In selected cases with persistent diagnostic uncertainty, a brain biopsy may be necessary. The presence of oligoclonal bands supports an inflammatory demyelinating process [4,9]. In our case, concordant imaging findings and CSF results were sufficient to support the diagnosis, and a brain biopsy was not performed. 

A limitation of this case is that advanced imaging techniques such as MR spectroscopy were not performed. Although conventional MRI features and CSF findings strongly supported a demyelinating process, MR spectroscopy could have provided additional metabolic information to further differentiate tumefactive demyelination from neoplastic or infectious etiologies.

High-dose corticosteroids remain first-line therapy for TMS, with symptom improvement reported in 60 to 80% of cases [10,11]. However, steroid resistance can occur in 20 to 30% of patients and may require escalation to plasmapheresis or other immunotherapies, including rituximab in selected cases [11]. In our patient, rapid clinical improvement after corticosteroid therapy is consistent with evidence that early treatment can reduce edema and accelerate lesion regression [10]. Although rapid clinical improvement after corticosteroid therapy supports an inflammatory demyelinating process, it is not diagnostic, as certain neoplastic conditions, such as primary CNS lymphoma, may also show transient clinical or radiographic improvement following steroid administration. Therefore, the diagnosis in this case relied on the combination of characteristic MRI findings, concurrent spinal demyelination, and cerebrospinal fluid oligoclonal bands rather than treatment response alone. While disease-modifying therapies are standard for relapsing-remitting multiple sclerosis (MS), their role after a first TMS episode remains debated. Available evidence supports considering disease-modifying therapy in patients with recurrence or evolution to conventional multiple sclerosis [11]. Disease-modifying therapy was not initiated, given the monophasic presentation, but it would be considered in the event of recurrent demyelinating episodes or if diagnostic criteria for multiple sclerosis are met in the future.

## Conclusions

Tumefactive multiple sclerosis (TMS) remains a rare and diagnostically challenging variant of multiple sclerosis that requires a high index of suspicion and careful integration of clinical, radiological, and laboratory findings. Distinguishing TMS from important mimics such as neoplasms, abscesses, and lymphoma is essential, with MRI features (including incomplete ring enhancement and relatively limited mass effect) and CSF findings such as oligoclonal bands playing a central role in diagnosis. TMS should be considered even in less typical demographic groups to avoid diagnostic delay and unnecessary invasive procedures. A multidisciplinary approach involving neurology, radiology, and infectious disease teams is often valuable when overlapping clinical features are present. Further research is needed to refine diagnostic approaches, validate emerging biomarkers, and clarify the role of disease-modifying therapies in recurrence prevention and long-term outcomes.
